# Temporal distribution and insecticide resistance profile of two major arbovirus vectors *Aedes aegypti* and *Aedes albopictus* in Yaoundé, the capital city of Cameroon

**DOI:** 10.1186/s13071-017-2408-x

**Published:** 2017-10-10

**Authors:** Basile Kamgang, Aurelie P. Yougang, Micareme Tchoupo, Jacob M. Riveron, Charles Wondji

**Affiliations:** 1 Research Unit Liverpool School of Tropical Medicine/Organisation de Coordination pour la lutte contre les Endémies en Afrique Centrale, P.O. Box 288, Yaoundé, Cameroon; 2 Department of Animal Biology, Faculty of Sciences, University of Yaoundé I, Yaoundé, Cameroon; 3 Liverpool School of Tropical Medicine, Pembroke place, Liverpool, L3 5QA UK

**Keywords:** *Aedes aegypti*, *Aedes albopictus*, Arboviruses, Temporal distribution, Spatial distribution, Insecticide resistance

## Abstract

**Background:**

*Aedes aegypti* and *Ae. albopictus* are the major epidemic vectors of several arbovirus diseases such as yellow fever, dengue, Zika and chikungunya worldwide. Both *Aedes* vectors are presents in Cameroon; however, knowledge on the dynamic of the distribution of these species across cities and their resistance profile to insecticide are limited. Here, we assessed the current distribution of *Ae. aegypti* and *Ae. albopictus* in Yaoundé, the Capital City, established the resistance profile to insecticides and explored the resistance mechanisms involved.

**Methods:**

Immature stages of *Aedes* were sampled in several breeding sites in December 2015 (dry season) and June 2016 (rainy season) in three central neighborhoods and four peripheral neighborhoods and reared to adult stage. The G0 adults were used for molecular identification and genotyping of F1534C mutation in *Ae. aegypti*. Bioassays and piperonyl butoxide (PBO) assays were carried out according to WHO guidelines.

**Results:**

Analysis revealed that both species *Ae. aegypti* and *Ae. albopictus* are present in all prospected sites in Yaounde. However, in the dry season *Ae. aegypti* is most abundant in neighborhoods located in downtown. In contrast, *Ae. albopictus* was found most prevalent in suburbs whatever the season and in downtown during the rainy season. Bioassay analysis showed that both *Ae. aegypti* and *Ae. albopictus*, are resistant to 0.05% deltamethrin, 0.1% bendiocarb and 4% dichlorodiphenyltrichloroethane (DDT). A decreased of susceptibility to 0.75% permethrin and a full susceptibility to malathion 5% was observed. The mortality rate was increased after pre-exposure to synergist PBO. None of *Ae. aegypti* assayed revealed the presence of F1534C mutation.

**Conclusion:**

These findings are useful to planning vector control programme against arbovirus vectors in Cameroon and can be used as baseline in Africa where data on *Aedes* resistance is very scarce to plan further works.

**Electronic supplementary material:**

The online version of this article (10.1186/s13071-017-2408-x) contains supplementary material, which is available to authorized users.

## Background

Yellow fever virus, dengue virus (DENV), chikungunya virus (CHIKV) and Zika virus (ZIKV) are mosquito-borne viruses of medical concern in tropical and subtropical regions. In Africa, until recently, the situation seemed to be of little concern because outbreaks of dengue without haemorrhagic syndromes had been observed only in East Africa [1, 2]. However, during the past decade, dengue outbreaks have been reported in several West and Central African countries [3–6] suggesting a possible change in the dynamic of this disease. Similarly, CHIKV, which previously caused only sporadic epidemics in sub-Saharan Africa [7], has recently emerged in several urban epidemic foci in Central Africa [6]. Formerly, sporadic isolation of ZIKV has been documented in human and mosquitoes in Asia and Africa [8]. But since the epidemic reported in Micronesia in 2007 [9], the geographical distribution has been expanded in the Americas where a massive outbreak has been reported [10].

*Aedes aegypti* Linneaus and *Ae. albopictus* (Skuse) are the main epidemic vectors of these viruses worldwide [11–13]. Both species are established in sub-Saharan Africa, where *Ae. aegypti* is native [14]. *Aedes albopictus* originated from Asia [15], has invaded all the five continents during the past three decades [16]. This species has been first reported in Central Africa in Cameroon in early 2000s, and since then has invaded almost all central African countries [6, 17]. Alarmingly, the introduction of *Ae. albopictus* in Central Africa coincided with the emergence of DENV, ZIKV and CHIKV in urban areas [6].

In Central Africa, *Ae. aegypti* and *Ae. albopictus* are found sympatric in several locations, notably in south Cameroon [18]. Both species are found in rural and urban areas where they breed in domestic (e.g. water storage and flower pots), peri-domestic (e.g. discarded tanks and used tyres) and natural (e.g. tree holes and plant axils) breeding sites [17, 18]. As there is still no vaccine or specific treatment for these viruses, vector control remains the cornerstone of prevention and outbreak control. The conventional strategies for controlling *Aedes* species are based on reduction of breeding sites and on insecticide-based interventions. Indeed, in emergency situations, space spraying with adulticides can reduce the density of adult mosquitoes [19]. Unfortunately, many vector control programmes are threatened by the development of insecticide resistance in *Ae. aegypti* and *Ae. albopictus* [20, 21]. Two major resistance mechanisms have been found involved on insecticide resistance: insensitivity target sites and increase in the rate of insecticide metabolism [22, 23]. Target site resistance is caused by mutations in target genes such as the voltage gated sodium channel (VGSC) causing knockdown resistance (*kdr*), mutations in the acetylcholinesterase (*Ace-1*) gene and GABA receptors [23, 24]. One of the most important target site resistance for mosquitoes is *kdr* as it confers resistance to both pyrethroids and dichlorodiphenyltrichloroethane (DDT). Several *kdr* mutations have been identified in *Ae. aegypti*, and the association between the V1016G/I and the F1534C mutations and pyrethroid resistance has been established [25–27]. *kdr* mutation is less prevalent in *Ae. albopictus* with only the mutation F1534C that has been detected [28]. However metabolic resistance through upregulation of detoxification genes is a major resistance mechanism in both species. The three main enzyme families responsible for insecticide resistance in mosquitoes are the monooxygenases (cytochrome P450s), glutathione S-transferases (GSTs) and carboxylesterases (COEs) [29, 30]. In Africa, data on ecological characterization of *Aedes* vectors as well as their insecticide resistance profiles are scarce. In the context of emerging arboviruses in numerous countries across the world and in Africa particularly, this study was performed to assess the current spatio-temporal distribution of *Ae. aegypti* and *Ae. albopictus*in in Yaoundé, the capital city of Cameroon, and the insecticide resistance profile as well as exploring the resistance mechanism involved.

## Methods

### Mosquito collection

**Fig. 1:**
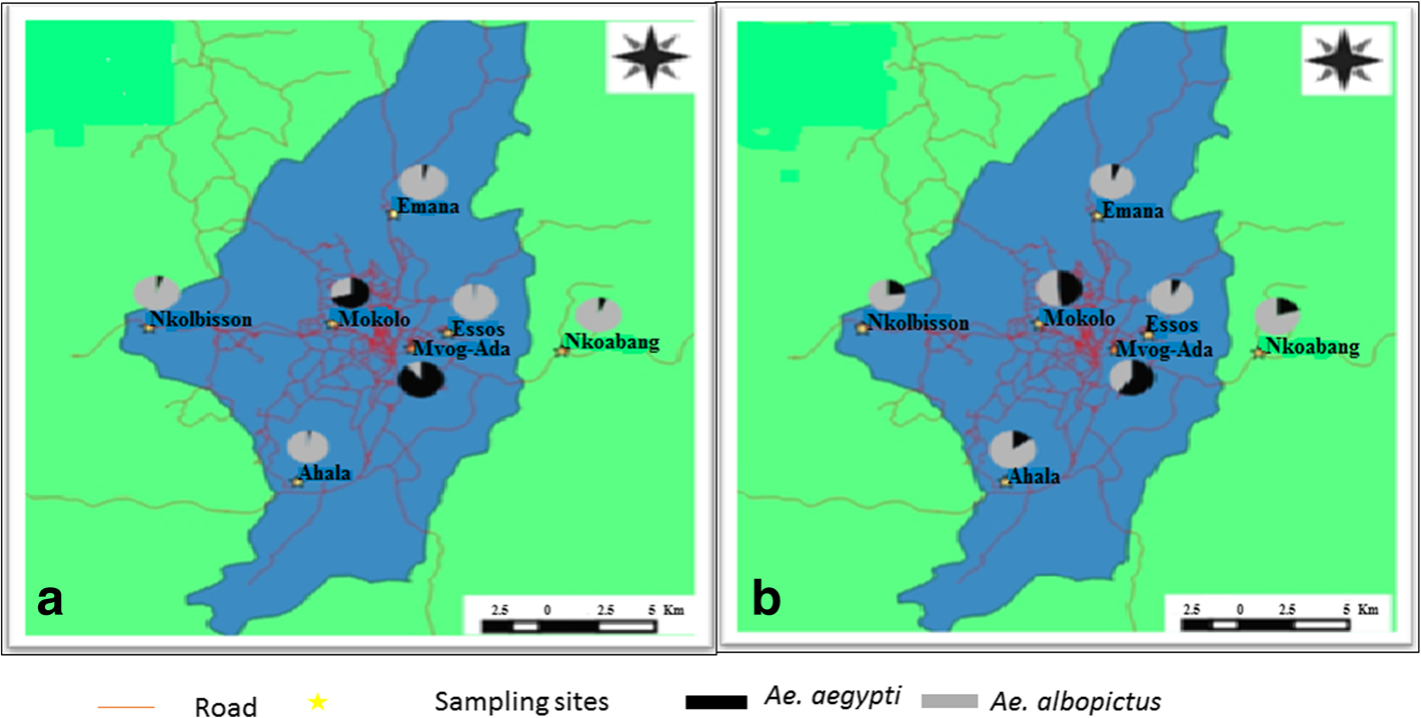
Sampling sites and geographical distribution of *Ae. aegypti* and *Ae. albopictus* in Yaoundé according to the season. **a** Dry season. **b** Rainy season

Immature stages of *Ae. aegypti* and *Ae. albopictus* were sampled in December 2015 (dry season) and June 2016 (rainy season) in seven neighborhoods in Yaoundé (Fig. 1). Based on a previous study in Yaoundé showing that *Ae. albopictus* seem to be most prevalent in peripheral areas of the city while *Ae. aegypti* is predominant in the city center [31], we selected three central neighborhoods (Mokolo, Mvog-Ada and Essos) and four peripheric (Nkolbisson, Emana, Ahala and Nkoabang). In each selected neighborhood, all potential larval breeding sites were inspected and positive sites (with at least one *Aedes* larvae or pupae) recorded. Immature stages of *Aedes* were collected, transported to the insectaries, pooled according to the location and reared to adult stage for identification. Adult mosquitoes were morphologically identified [32], numbered, pooled in a breeding cage according to species and location and further reared in the controlled condition (27 ± 2 °C; relative humidity 80 ± 10%) until generation 1 (G1) or subsequent G2 and G3. The comparison between the prevalence of *Ae. aegypti* and *Ae. albopictus* has been performed using chi-square tests.

### Adult insecticide bioassays

*Aedes aegypti* and *Ae. albopictus* bioassays were performed with non-blood-fed females according to the standard WHO guidelines [33]. Two reference susceptible strains were used as controls: the *Ae. aegypti* New Orleans strain and the *Ae. albopictus* susceptible strain from the Malaysia Vector Control Research Unit. Insecticide-impregnated papers were supplied by Liverpool School of Tropical Medicine. Two to five day-old F3 generation of *Aedes* mosquitoes from dry season and F1 generation from raining season with 4 replicates of 25 mosquitoes per tube were tested. The insecticides tested were: 0.75% permethrin (type I pyrethroid), 0.05% deltamethrin (type II pyrethroid), 4% DDT (organochlorine), 0.1% bendiocarb (carbamate) and 5% malathion (organophosphate). Mortality was recorded after 24 h and survivors were stored at -80 °C whereas dead mosquitoes were kept in silica gel into 1.5 ml tubes.

### Synergist assay with piperonyl butoxide

In order to investigate the potential role of oxidase-specific metabolic resistance mechanisms, synergist assays with piperonyl butoxide (PBO) was performed. Adult 2–5 day-old mosquitoes were pre-exposed to papers impregnated with 4% PBO for one h and then immediately exposed to three insecticides, DDT, deltamethrin and bendiocarb, for which higher level of resistance has been observed. Mortality was recorded after 24 h and compared to the results obtained with each insecticide without PBO and to a control sample exposed only to PBO.

### F1534C genotyping using allele specific PCR in *Aedes aegypti*

Genomic DNA was extracted for 59 and 83 F0 individuals of *Ae. aegypti* and *Ae. albopictus*, respectively, from two locations, using the Livak method [34]. To confirm morphological identification, molecular identification was carried out using a polymerase chain reaction (PCR) based method described by Beebe et al. [35] to differentiate between *Ae. aegypti* and *Ae. albopictus*. All specimens confirmed as *Ae. aegypti* were used to genotype the F1534C mutation known to be associated to DDT and pyrethroid resistance in *Ae. aegypti* in several countries worldwide including Africa such as in Ghana (West Africa) [36]. This mutation was genotyped using the allele specific PCR method as previously described [37].

## Results

### Relative abundance and spatial distribution of *Ae. aegypti* and *Ae. albopictus*

A total of 4971 immature specimens of *Aedes* spp. were collected in December 2015 (dry season) and June 2016 (rainy season) in seven neighborhoods of the Yaoundé city. *Aedes albopictus* was found more prevalent (74.2%) than *Ae. aegypti* (25.8%). Analyses performed according to locations (suburbs vs downtown) and seasons revealed that in dry season *Ae. aegypti* is most abundant in neighborhoods located in downtown such as Mokolo and Mvog-Ada (Table 1). In contrast, *Ae. albopictus* was found most prevalent in suburbs whatever the season and in downtown during the rainy season (Table 1). No significant difference was found between overall numbers of *Ae. albopictus* and *Ae. aegypti* collected in downtown during the dry season (*χ* ^2^ = 2.25, *df* = 2, *P* > 0.2), while the number of *Ae. albopictus* was significantly higher than those of *Ae. aegypti* (*χ* ^2^ = 1125.96, *df* = 2, *P* < 0.001) in suburbs during the dry season and in rainy season irrespective to the location (Table 1). In suburbs, the number of *Ae. aegypti* was lower than the number of *Ae. albopictus* in each location independently to the season (Table 1). In the other hand, in downtown the abundance of *Ae. aegypti* was higher than that of *Ae. albopictus* (*χ* ^2^ = 369.05, *df* = 2, *P* < 0.001) in Mokolo and (*χ* ^2^ = 240.25, *df* = 2, *P* < 0.001) Mvog-Ada in the dry season whereas in the rainy season *Ae. albopictus* was more abundant than *Ae. aegypti* in Essos (*χ* ^2^ = 295.81, *df* = 2, P < 0.001). The assessment of the spatial distribution of these two species in Yaoundé showed that both *Aedes* species *Ae. aegypti* and *Ae. albopictus* coexist in all the prospected areas of the city (Fig. 1, Table 1).

**Table 1:** Relative abundance of *Ae. aegypti* and *Ae. albopictus* from several locations in Yaoundé according to season

Location	Dry season				Rainy season			
	Breeding sites^a^	*Ae. aegypti*	*Ae. albopictus*	*P*	Breeding sites^a^	*Ae. aegypti*	*Ae. albopictus*	*P*
Downtown	21	509	544	< 0.1	29	492	731	< 0.001
Mokolo	Used tires (10); Car wrecks (1)	470	199	< 0.001	Used tires (5)	178	192	> 0.05
Mvog-ada	Used tires (2)	35	4	< 0.001	Used tires (11)	289	195	< 0.001
Essos	Used tires (8)	4	341	< 0.001	Used tires (12); Discarded tanks (1)	25	344	< 0.001
Surbub	50	49	1222	< 0.001	78	232	1192	< 0.001
Emana	Used tires (8)	10	241	< 0.001	Used tires (13); Cobblestone moulds (30)	37	491	< 0.001
Nkolbisson	Used tires (10)	28	549	< 0.001	Used tires (9); Discarded tanks (1); Rubber boot (1)	146	480	< 0.001
Ahala	Used tires (10); Discarded tanks (5)	9	404	< 0.001	Used tires (9)	19	105	< 0.001
Nkoabang	Used tires (16)	2	28	< 0.001	Used tires (15)	30	116	< 0.001

^a^Numbers in parentheses indicate the number of breeding sites with immature stages of *Aedes*

### Insecticide resistance profiles

Tests performed with laboratory strains confirmed that *Ae. albopictus* (VCRU) and *Ae. aegypti* (New Orleans) were totally susceptible to insecticides tested except to DDT for which 80.68% and 98.75% mortality rates were found, respectively. The mortality rate in controls was inferior to 5%.

#### Resistance pattern for *Aedes aegypti*

**Fig. 2:**
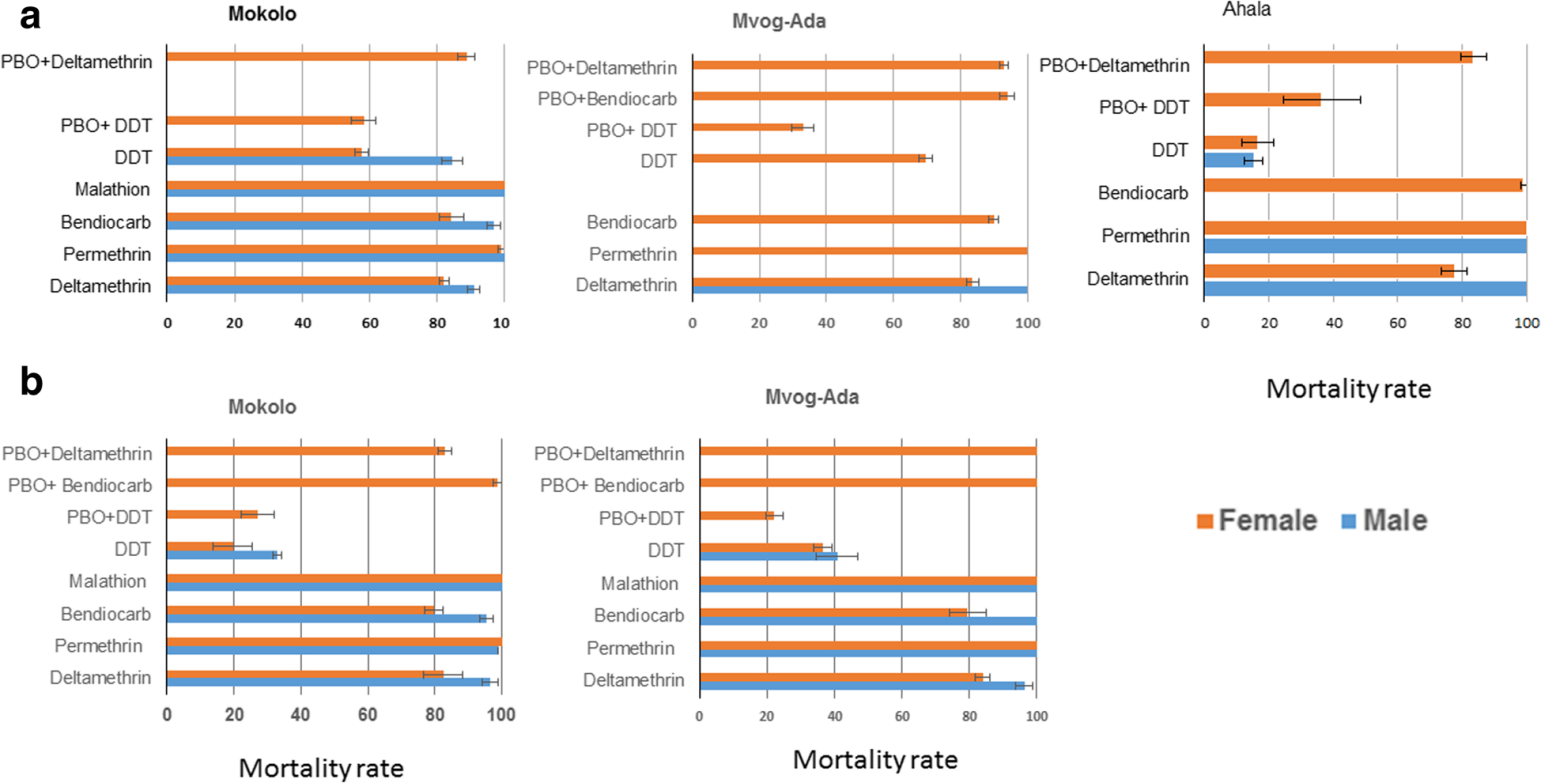
Mortality rates of adult *Ae. aegypti* from Yaoundé neighbourhoods 24 h after exposure to insecticides alone or with 1 h pre-exposure to PBO. **a** Rainy season. **b** Dry season. Error bars represent standard error of the mean

The two populations collected during the dry season were first tested revealing that both were resistant to the type II pyrethroid, deltamethrin, particularly in females with similar mortality rates ranging from 82.42% in Mokolo to 83.90% in Mvog-Ada (Additional fie 1: Table S1). In contrast, both populations were fully susceptible to type I pyrethroid, permethrin. Assays with the carbamate, bendiocarb, revealed that both populations were resistant to this insecticide particularly in females with similar mortality of 79.78 and 79.52% in Mokolo and Mvog Ada, respectively (Additional file 1: Table S1, Fig. 2). The highest level of resistance was observed against DDT with both populations resistant with mortality rates ranging from 19.57 to 36.47% in Mokolo and Mvog-Ada, respectively for females (Additional file 1: Table S1, Fig. 2). However, both populations were fully susceptible to the organophosphate malathion.

Bioassays performed during the raining season were overall consistent with the results obtained during the dry season apart for DDT for which an increased mortality was observed in the raining season in Mokolo (19.57–57.65%; *χ* ^2^ = 25.1, *df* = 2, *P* < 0.001) and Mvog-Ada (36.47–69.62%; *χ* ^2^ = 15.7, *df* = 2, *P* < 0.001). The third population of Ahala tested in the wet season also showed similar resistance profiles to the other two. However, the Ahala population was fully susceptible to bendiocarb and exhibited a greater resistance to DDT (16.48%) than the other two populations (Additional file 1: Table S1, Fig. 2).

#### *Aedes albopictus* resistance pattern

**Fig. 3:**
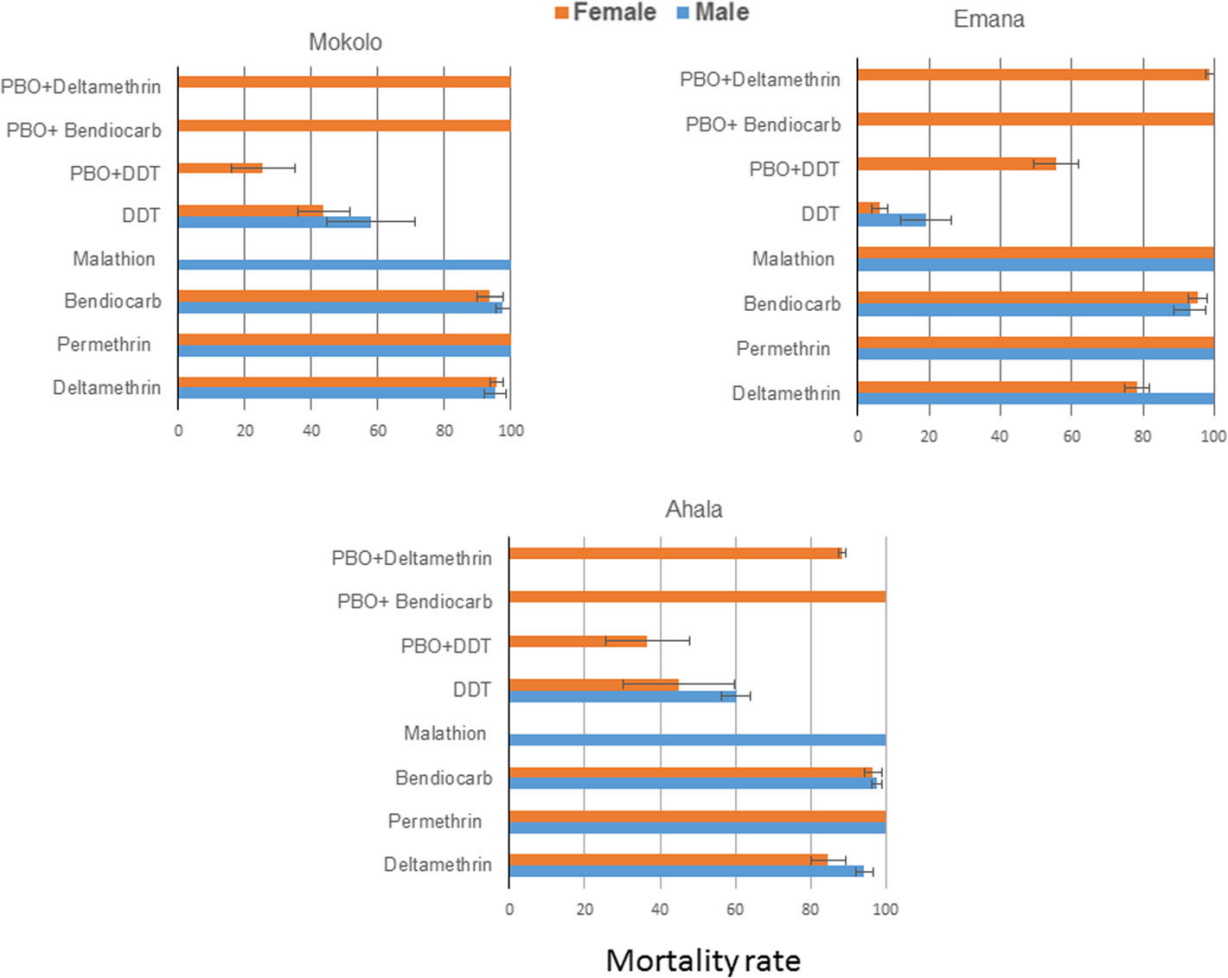
Mortality rates of adult *Ae. albopictus* in the dry season from Yaoundé neighbourhoods 24 h after exposure to insecticides alone or with 1 h pre-exposure to PBO. Error bars represent standard error of the mean

Bioassays performed during the dry season revealed that three populations tested were resistant to the type II pyrethroid, deltamethrin although in Mokolo this was only moderate with 95.8% in females (Fig. 3, Additional file 2: Table S2). As for *Ae. aegypti*, a full susceptibility was observed against the type I permethrin and also against the organophosphate malathion in all populations. A moderate resistance was observed against the carbamate bendiocarb with mortality rates ranging between 93.1–95.5% in females. Also similar to *Ae. aegypti*, high resistance levels were observed against DDT in all three populations with mortality rates ranging between 5.9–44.8% in females (Fig. 3, Additional file 2: Table S2).

**Fig. 4:**
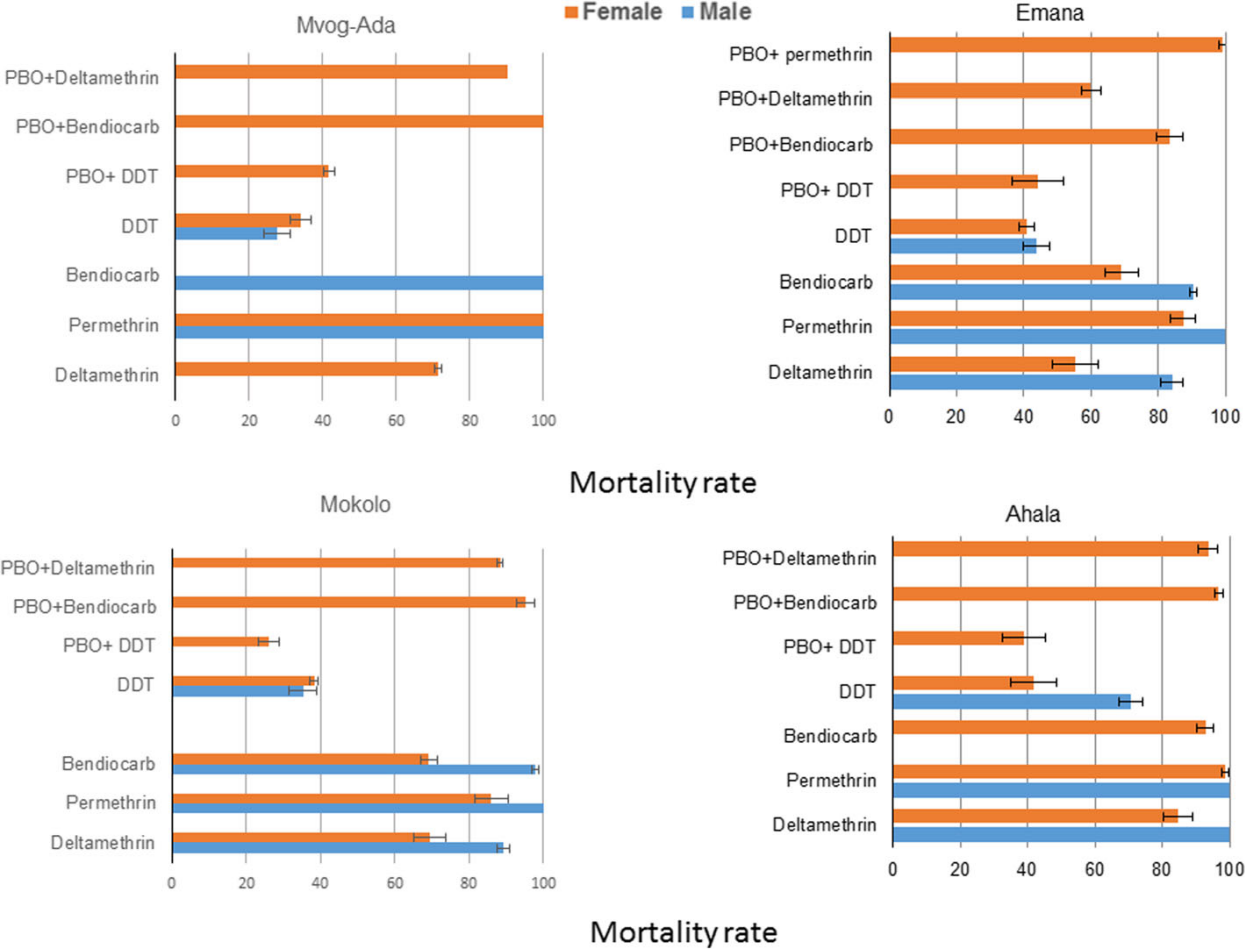
Mortality rates of adult *Ae. albopictus* collected in the rainy season from Yaoundé neighbourhoods 24 h after exposure to insecticides alone or with 1 h pre-exposure to PBO. Error bars represent standard error of the mean

The samples collected during the wet season exhibited a greater resistance levels for several insecticides (Fig. 4). This includes deltamethrin for which a decrease in mortality rates was observed in two populations from 95.8 to 69.3% in Mokolo (*χ* ^2^ = 10.13, *df* = 2, *P* < 0.005) and 78.3 to 55.2% in Emana (*χ* ^2^ = 9.66, *df* = 2, *P* < 0.005) (Fig. 4, Additional file 2: Table S2). Similarly, for bendiocarb, mortality rates decreased from 93.8 to 69.2% in Mokolo (*χ* ^2^ = 10.13, *df* = 2, *P* < 0.005) and from 95.2 to 69% in Emana (*χ* ^2^ = 9.94, *df* = 2, *P* < 0.005) but no significant change was observed in Ahala. The Mokolo and Emana populations were resistant to permethrin in the wet season with mortality rates of 86 and 87.3%, respectively. While 100% of mortality were found in dry season (Additional file 2: Table S2) event if no significant difference was observed on mortality rate between both seasons).

### Synergist assays with PBO

Pre-exposure of mosquito samples to the PBO synergist before bioassays to deltamethrin, DDT and bendiocarb revealed contrasting results with both cases of increased or unchanged mortality rates in both *Ae. aegypti* (Additional file 1: Table S1) and *Ae. albopictus* (Additional file 2: Table S2) with deltamethrin and bendiocarb during both dry and wet seasons. One example is the case of the *Ae. aegypti* population from Emana during the dry season which showed a recovery of susceptibility from 83.9 to 100% after PBO exposure. However other populations did not show a significant increase in mortality after PBO such as the *Ae. aegypti* populations from Mokolo (82.4% mortality without PBO and 82.6% after PBO exposure). On the other hand, no increase of mortality was observed with DDT in *Ae. aegypti* from Mvog-Ada (Additional file 1: Table S1) and *Ae. albopictus* from Mokolo and Ahala (Additional file 2: Table S2).

### Genotyping of F1534C mutation in *Aedes aegypti*

59 F0 specimens of *Ae. aegypti* and 83 F0 of *Ae. albopictus* identified morphologically were confirmed by molecular analysis. All the specimens of *Ae. aegypti* were used to search the presence of F1534C mutation. None of the *Ae. aegypti* mosquitoes was found carrying this mutation.

## Discussion

This study presents the current distribution of *Ae. aegypti* and *Ae. albopictus* in Yaoundé, the capital city of Cameroon and the resistance profile of these two species. The results revealed that both species are present in all the prospected locations of the city and *Ae. albopictus* is predominant during the two collection periods. Bioassay experiments revealed that both species are resistant to DDT and deltamethrin, loss of sensitivity was noticed to permethrin and bendiocarb, and fully susceptibility to malathion.

### Temporal distribution of both species

The predominance of *Ae. albopictus* in both dry and rainy seasons is inconsistent with previous observations made in the Central African Republic [17] and in Florida, USA [38] showing the predominance of *Ae. aegypti* at the early wet season and *Ae. albopictus* in the late wet season. These findings had been explained by the higher tolerance of *Ae*. *aegypti* eggs than those of *Ae. albopictus* as demonstrated by Lounibos et al. [39]. The difference observed between these previous studies and the current is due probably to the difference of time between the rainy season and the dry season in these locations. The predominance of the invasive species *Ae. albopictus* in Yaoundé is in agreement with the previous observations made in the city [31] suggesting that *Ae. albopictus* tends to supplant the indigenous species *Ae. aegypti*. This observation suggests a good adaptation of *Ae. albopictus* due to the high ecological plasticity of this species, allowing it to adapt in different environments and to mating interference in sympatric areas as demonstrated by Bargielowski et al. [40]. *Aedes aegypti* was found dominant in the downtown particularly during the dry season such as Mokolo and Mvog-Ada whereas *Ae. albopictus* is the most abundant species in the suburbs. These findings are consistent with previous results showing segregation of both species according to urbanization in sympatric area [38]. Nevertheless, *Ae. albopictus* was found more abundant during the rainy season in Mokolo (downtown neighborhood). This difference between both seasons is probably due to the environmental change notably destroyed buildings due to regeneration project in Mokolo which has allowed the proliferation of vegetation which is very favourable to the development of *Ae. albopictus* as demonstrated previously [17, 31].

The higher number of immature stage of *Aedes* spp. collected in the rainy season is in agreement with previous observations showing that rainy season corresponds to the period in which the maximum densities of *Aedes* spp. mosquitoes are observed [17], suggesting higher risk of arboviruses transmission. However, in some neighborhoods fewer mosquitoes were collected in the wet season, such as the case of Ahala and Mokolo, potentially due to water overflow from some breeding sites which could have drained larvae from their habitats.

### Insecticide resistance patterns between both species

Overall, this study revealed that both species of *Aedes* present a similar resistance profile to main insecticides used in public health. Indeed, both species are resistant to 0.05% deltamethrin, 0.1% bendiocarb (carbamate) and 4% DDT and fully susceptible to 5% malathion. Almost all samples were found susceptible to 0.75% permethrin except two samples from Mokolo and Emana collected during the rainy season. This result is different to a previous study assessing the insecticide resistance profile of *Ae. aegypti* and *Ae. albopictus* in four cities of Cameroon [41]. This previous study had revealed that *Ae. aegyti* from Yaoundé was fully susceptible to four insecticides tested (deltamethrin, DDT, propoxur (carbamate) and fenitrothion (organophosphate) while *Ae. albopictus* was resistant to deltamethrin and DDT suggesting that both species have developed resistance to most of these insecticide classes in the past five years. However, a striking difference was observed with pyrethroids with higher resistance in deltamethrin (type II) whereas both species were more susceptible to permethrin (type I). Such differences have been previously observed in other populations of mosquitoes such as in Malaysia where an *Ae. aegypti* population from Kota Bharu was highly resistant to permethrin but not to deltamethrin [42, 43]. On the other hand, the low level of resistance reported to permethrin in these *Aedes* spp. populations could also be explained by the fact that the dose used in this study (0.75%) is three-fold higher than what is recommended for *Aedes* species (0.25%) [44]. It will be necessary to test these populations with this lower concentration of permethrin to establish the real resistance level. The high level of resistance observed in *Ae. albopictus* compared to that of *Ae. aegypti* is different from previous findings showing that *Ae. aegypti* is more resistant to pyrethroids than *Ae. albopictus* [42, 45]*.* Nevertheless, similar observations have been reported in previous studies in Central Africa, particularly in Cameroon [41] and the Central African Republic [46]. The decreased susceptibility to both types of pyrethroids observed in both populations may represent a serious threat for vector control programmes, since pyrethroids only are recommended for the control of adult *Aedes* mosquitoes notably in emergency situations [47, 48]. A loss of sensitivity was observed to bendiocarb for both species. Similar results have been observed previously in Pakistan and Malaysia [42, 49].

Both *Ae. aegypti* and *Ae. albopictus* samples from all locations were found resistant to DDT. Previous study in 1972 has reported decreased susceptibility to DDT in *Ae. aegypti* sampled in Yaoundé [50], suggesting continuing selection pressure on *Aedes* spp. populations. DDT-resistance to *Ae. albopictus* has also been reported in Yaoundé samples [41]. High DDT resistance in *Ae. aegypti* [36, 42, 46, 51] and *Ae. albopictus* [41, 42, 45, 52] is commonly reported across the world. The higher level of DDT resistance in both species in Yaoundé is also similar to high resistance level to DDT observed in Cameroon in malaria vectors such as *Anopheles gambiae* [53] or *An. funestus* [54] probably as a consequence of the intense DDT spraying in the 1950s and 1960s as part of the malaria elimination campaign.

The causes of the resistance to pyrethroids and to bendiocarb in both species remains unclear since no specific vector control interventions targeting *Ae. aegypti* and *Ae. albopictus* has been deployed [41]. Nevertheless, it is possible that insecticides used to control other insects of medical or agricultural interest exert indirect selection pressure on these two mosquito species as suggested previously [41, 46]. For *Ae. albopictus*, which was reported for the first time in Cameroon in the early 2000s, we cannot exclude the possibility that the invading population possessed a resistance background as suggested previously [41]. A temporal variation of the resistance profile was observed with high resistance during the second collection (rainy season) especially in *Ae. albopictus* samples particularly from Mokolo and Emana. The increasing of the level of resistance in the rainy season remains unclear.

Increasing of mortality rates in both *Ae. aegypti* and *Ae. albopictus* with deltamethrin and bendiocarb after pre-exposure to PBO suggest that cytochrome P450 monooxygenases are playing a predominant role in the observed resistance. On the other hand, no increase of mortality observed with DDT in both species in certain locations and no full recovery of susceptibility suggest that other enzymes such as GSTs may also implicated. None of the specimens of *Ae. aegypti* genotyped was found with the presence F1534C mutation suggesting this mutation is not involved in resistance in the sample of *Ae. aegypti* tested. Nevertheless, this mutation was detected recently in *Ae. aegypti* sample from West Africa (Ghana) [36]. It will be interesting to extent this work in other locations throughout the country and also genotype other mutations such as I1011M/V, V1016G/I which have been found involved in *kdr* resistance in *Ae. aegypti* [25–27].

## Conclusion

This study has shown that the invasive *Ae. albopictus* is now the most dominant arbovirus vector in Yaoundé irrespective of the season. It was also found this species to be more resistant than the indigenous species *Ae. aegypti*. This could be a concern for the control of arboviruses as *Ae. albopictus* has been reported to be mostly involved in the recent dengue, Zika and chikungunya outbreaks in Central Africa. Overall, the findings of this study highlight the need for more studies nationwide to better characterize these arbovirus vectors and help prepare potential outbreaks.

## Additional files

Additional file 1: **Table S1.** Mortality rates of adult *Ae. aegypti* from Yaoundé neighbourhoods 24 h after exposure to insecticides alone or with 1 h pre-exposure to PBO. (DOC 47 kb)

Additional file 2: **Table S2.** Mortality rates of adult *Ae. albopictus* from Yaoundé neighbourhoods 24 h after exposure to insecticides alone or with 1 h pre-exposure to PBO. (DOC 52 kb)

## Abbreviations

DDT: dichlorodiphenyltrichloroethane
*kdr*: knock down resistance gene
PBO: piperonyl butoxide
PCR: polymerase chain reaction
