# Supplementary material for: Temporal distribution and insecticide resistance profile of two major arbovirus vectors Aedes aegypti and Aedes albopictus in Yaoundé, the capital city of Cameroon
Source: Parasit Vectors. 2017 Oct 10;10:469. doi: 10.1186/s13071-017-2408-x (PMC5635539; doi:10.1186/s13071-017-2408-x)
Supplement: Supplementary file 1 — Table S1. Mortality rates of adult Ae. aegypti from Yaoundé neighbourhoods 24 h after exposure to insecticides alone or with 1 h pre-exposure to PBO. (DOC 47 kb) [file 13071_2017_2408_MOESM1_ESM.doc]

**Table S1:** Mortality rates of adult *Aedes aegypti* from Yaoundé neighbourhoods 24 h after exposure to insecticides alone or with 1 h pre-exposure to PBO.

NA. Not available; PBO, pyperonyl butoxide.

| Insecticides | Mortality rate ± standard error ( no. of mosquitoes assayed) | | | | | |
| --- | --- | --- | --- | --- | --- | --- |
|  | ***Dry season*** | | | | | |
|  | Mokolo | | Mvog-Ada | | Ahala | |
|  | Male | Female | Male | Female | Male | Female |
| 0.05% Deltamethrin | 96.43±2.29  (84) | 82.42±5.89  (91) | 96.34±2.51  (82) | 83.90±2.22  (87) | NA | NA |
| 0.75% Permethrin | 98.70±1.25 (77) | 100±0  (87) | 100±0  (82) | 100±0  (81) | NA | NA |
| 0.1% Bendiocarb | 95.51±2.05 (84) | 79.78±2.71 (89) | 100±0  (87) | 79.52±5.28 (83) | NA | NA |
| 5% Malathion | 100±0 (88) | 100±0  (90) | 100±0  (85) | 100±0  (82) | NA | NA |
| 4% DDT | 32.91±1.26 (79) | 19.57±6 (92) | 40.74±6.14 (81 | 36.47±2.70 (85) | NA | NA |
| 4% PBO + 4% DDT | NA | 27.06±4.81 (85) | NA | 22.07±2.51 (77) | NA | NA |
| 4%PBO+0.1%Bendiocarb | NA | 98.70±1.25 (77) | NA | 100±0  (78) | NA | NA |
| PBO + Deltamethrin | NA | 82.86±2.04 (70) | NA | 100±0  (85) | NA | NA |
|  | ***Rainy season*** | | | | | |
| 0.05% Deltamethrin | 90.91±2.08  (99) | 82.05±4.22  (78) | 100±0  (94) | 83.52±6.36  (91) | 100±0  (96) | 77.53±3.87  (89) |
| 0.75%Permethrin | 100±0  (85) | 98.98±1.04 (98) | NA | 100±0  (86) | 100±0  (97) | 100±0  (100) |
| 0.1% Bendiocarb | 96.91±1.06  (97) | 84.27±6.42 (89) | NA | 89.80±1.19 (98) | NA | 99±0.96  (101) |
| 5% Malathion | 100±0  (89) | 100±0  (80) | NA | NA | NA | NA |
| 4% DDT | 84.34±4.41  (83) | 57.65±3.35 (85) | NA | 69.62±5.22 (79) | 15.15±2.94 (66) | 16.48±4.81  (91) |
| 4% PBO + DDT | NA | 58.23±7.99 (79) | NA | 32.93±10.02 (82) | NA | 36.47±12.03 (85) |
| PBO +Bendiocarb | NA | NA | NA | 93.88±2.33 (98) | NA | NA |
| PBO+Deltamethrin | NA | 88.75±5.33 (80) | NA | 92.94±2.88 (85) | NA | 83.52±3.98 (91) |
