# Supplementary material for: Temporal distribution and insecticide resistance profile of two major arbovirus vectors Aedes aegypti and Aedes albopictus in Yaoundé, the capital city of Cameroon
Source: Parasit Vectors. 2017 Oct 10;10:469. doi: 10.1186/s13071-017-2408-x (PMC5635539; doi:10.1186/s13071-017-2408-x)
Supplement: Supplementary file 2 — Table S2. Mortality rates of adult Ae. albopictus from Yaoundé neighbourhoods 24 h after exposure to insecticides alone or with 1 h pre-exposure to PBO. (DOC 52 kb) [file 13071_2017_2408_MOESM2_ESM.doc]

**Table S2**: Mortality rates of adult *Aedes albopictus* from Yaoundé neighbourhoods 24 h after exposure to insecticides alone or with 1 h pre-exposure to PBO.

| Insecticides | Mortality rate ± standard error ( no. of mosquitoes assayed) | | | | | | | |
| --- | --- | --- | --- | --- | --- | --- | --- | --- |
|  | Dry season | | | | | | | |
|  | Mokolo | | Emana | | Ahala | | Mvog-Ada | |
|  | Male | Female | Male | Female | Male | Female | Male | Female |
| Deltamethrin 0.05% | 95.3±3  (86) | 95.8±1.8 (97) | 100±0  (86) | 78.3±3.3 (83) | 94.2±2.2 (87) | 84.6±4.6 (78) | NA | NA |
| Permethrin 0.75% | 100±0  (93) | 100±0  (89) | 100±0  (86) | 100±0  (84) | 100±0  (85) | 100±0  (86) | NA | NA |
| Bendiocarb 0.1% | 97.7±2 (88) | 93.8±3.4 (81) | 93.1±4.3 (88) | 95.2±2.6 (85) | 97.6±1.2 (86) | 96.5±2.2 (86) | NA | NA |
| Malathion 5% | 100±0  (97) | NA | 100±0  (82) | 100±0  (86) | 100±0  (86) | NA | NA | NA |
| DDT 4% | 57.8±13.2 (102) | 43.5±7.8 (78) | 19.2±7 (83) | 5.9±2.2 (84) | 60.2±3.8 (83) | 44.8±14.7 (107) | NA | NA |
| PBO 4%+ DDT | NA | 25.4±9.6 (59) | NA | 55.5±6.4 (90) | NA | 36.7±11.3 (79) | NA | NA |
| PBO +Bendiocarb | NA | 100±0  (62) | NA | 100±0 (86) | NA | 100±0  (82) | NA | NA |
| PBO+Deltamethrin | NA | 100±0  (91) | NA | 98.7±1.1 (82) | NA | 88.4±1  (78) | NA | NA |
|  | ***Rainy season*** | | | | | | | |
| Deltamethrin 0.05% | 89.2±3.5  (84) | 69.3±7 (88) | 84±3.2  (99) | 55.2±6.6  (85) | 100±0  (80) | 84.7±4.4 (92) | NA | 71.4±1.2  (81) |
| Permethrin 0.75% | 100±0  (100) | 86±5.3 (86) | 100±0  (99) | 87.3±3.6  (87) | 100±0  (79) | 98.9±1  (93) | 100±0  (84) | 100±0  (82) |
| Bendiocarb 0.1% | 97.9±1.1 (97) | 69.2±5.5 (91) | 90.3±1  (104) | 69±4.9  (97) | NA | 92.7±2.6 (97) | 100±0  (100) | 98±1.1  (102) |
| DDT 4% | 35.2±1.8 (88) | 38.2±0.6 (81) | 43.6±3.7  (87) | 40.7±2.3  (86) | NA | 41.7±6.7 (79) | 27.5±7  (87) | 34±7.8  (87) |
| PBO 4%+ DDT | NA | 25.9±6.8 (81) |  | 44.1±7.6  (86) | NA | 38.9±6.2  (77) | NA | 41.7±3.5  (67) |
| PBO +Bendiocarb | NA | 95.3±2 (86) | NA | 83.1±3.9  (89) | NA | NA | NA | 100±0  (100) |
| PBO+Deltamethrin | NA | 88.3±0.4 (86) | NA | 60±3  (90) | NA | 93.6±2.7 (63) | NA | 90.3±3.1  (83) |
| PBO+ permethrin | NA | NA | NA | 98.9±1.1  (95) | NA | 96.8±1.3 (63) | NA | NA |

NA. Not available; PBO, pyperonyl butoxide.
